# Current advances and challenges in microbiome-based mitigation of volatile organic compounds from livestock waste systems

**DOI:** 10.3389/fcimb.2026.1848842

**Published:** 2026-07-03

**Authors:** Mounir Adnane, Marc Drillich, Aspinas Chapwanya

**Affiliations:** 1 Department of Biomedicine, Institute of Veterinary Sciences, University of Tiaret, Tiaret, Algeria; 2 Unit for Reproduction Medicine and Udder Health, Faculty of Veterinary Medicine, Freie Universität Berlin, Berlin, Germany; 3 Department of Clinical Sciences, Ross University School of Veterinary Medicine, Basseterre, Saint Kitts and Nevis

**Keywords:** animal waste, livestock production systems, manure microbiome, microbiome engineering, odor mitigation, sustainable agriculture, volatile organic compounds

## Abstract

Odor emissions from animal waste represent a persistent challenge in livestock production, with implications for animal welfare, environmental quality, and the societal sustainability of farming. These emissions are primarily driven by volatile organic compounds (VOCs) generated through microbial degradation of feces and urine, including ammonia, sulfur-containing compounds, volatile fatty acids, and aromatic metabolites. Conventional odor control strategies rely largely on physical, chemical, or management-based approaches, which often provide inconsistent or short-term mitigation and raise concerns regarding cost and sustainability. Advances in microbiome research have highlighted the central role of gut-associated and manure-associated microbial communities in shaping VOC production, positioning microbiome engineering as a promising novel biological alternative for odor mitigation. This review synthesizes current knowledge on microbiome-based strategies targeting VOCs in animal waste, including dietary modification, probiotic and functional microbial consortia approaches, as well as post-excretion bioaugmentation. This review evaluates also the microbial basis, health and biosecurity relevance, and practical limitations of these mitigation strategies. While microbiome engineering shows considerable potential, its effectiveness remains highly context dependent, and broader adoption is constrained by variability across production systems and limited farm-scale validation. Future interventions will require mechanism-driven research, standardized methodologies, and integration within comprehensive waste management frameworks to support sustainable livestock production.

## 1 Introduction

The management of odors from animal waste presents a complex and persistent challenge to modern livestock production, with far reaching impacts varying from mere nuisance to animal welfare, occupational health, environmental quality, and the social sustainability of farming operations (Cao et al., 2023). The principal agents of these malodorous products are volatile organic compounds (VOCs) that are generated during microbial degradation of feces and urine. Key byproducts include ammonia (NH_3_), hydrogen sulfides (H_2_S), volatile fatty acids (VFAs), phenols, indole compounds (e.g., skatole), and volatile sulfur compounds (Le et al., 2007; Cao et al., 2023; Hwang et al., 2023). The emission of these molecules into the environment is exacerbated by intensive production systems for increased food production, where high stocking densities and protein-dense diets fed to animals promote microbial proteolysis and anaerobic fermentation (Abdugheni et al., 2023).

Conventional odor control strategies, including ventilation optimization, dietary protein reduction, chemical masking, and manure storage modifications, are often constrained by high costs, limited sustainability, and potential ecological disruption (Jin Song et al., 2019; Cao et al., 2023). Chemical interventions raise concerns regarding residues and the disruption of local ecologies (Jin Song et al., 2019; Park et al., 2024b).The microbial processes governing manure decomposition are therefore not only linked to odor and VOC emissions but also to bioenergy recovery efficiency. Consequently, interventions aimed at modifying microbial communities to reduce VOC emissions may also influence methanogenic activity and overall biogas production performance. Consequently, there is a growing interest in biologically driven, microbiome-centered strategies that address VOC emissions at their sources.

Recent advances in microbiome science and microbial ecology have reshaped our understanding of the generation of odor. This is no longer viewed merely as a passive outcome of waste accumulation, but rather as a functional result of structured, dynamic, and metabolically integrated microbial ecosystems operating within the animal gastrointestinal tract and in post-excretion waste environments (Thorn and Greenman, 2012; Abdugheni et al., 2023). Specific microbial taxa and metabolic pathways implicated in odorous VOC production in livestock waste include sulfate-reducing bacteria (e.g., *Desulfovibrio* spp.) generating H_2_S, proteolytic Clostridium and Bacteroides species producing NH_3_ and branched-chain fatty acids, and amino acid-fermenting bacteria such as *E. coli* and Fusobacterium that produce indoles and skatole (Karnachuk et al., 2021; Zhang et al., 2023; Omer et al., 2025). This insight positions the manure and gut microbiome at the cutting-edge and targetable components for next-generation odor mitigation strategies (Jin Song et al., 2019; Abdugheni et al., 2023).

Microbiome engineering, or the intentional manipulation of microbial community structure or function to achieve a desired outcome, has emerged as a promising framework for VOC reduction (Jin Song et al., 2019). Strategies include probiotic supplementation, prebiotic substrate modulation, targeted bioaugmentation with functional consortia, and the application of amendments like biochar to shape microbial habitats (Hwang et al., 2023; Park et al., 2024b; Frazier et al., 2025). Importantly, livestock species differ substantially in gastrointestinal physiology and microbial ecology, which influences the design and effectiveness of microbiome-based interventions. Ruminants rely on a complex foregut fermentation system in the rumen, where dense anaerobic microbial communities drive the degradation of fibrous substrates and generate fermentation products. In contrast, monogastric animals such as horses, pigs and poultry exhibit limited foregut fermentation, with microbial activity primarily occurring in the hindgut. Consequently, strategies involving oral administration of probiotics, prebiotics, or other microbiome-modulating compounds must account for these physiological differences, as microbial establishment, substrate availability, and metabolic outcomes vary across species. For instance, studies have shown that probiotic feed additives can shift the rumen microbial community structure in cattle, leading to significant reductions in emissions of ammonia and volatile fatty acids (Park et al., 2024b). Similarly, biochar amendments to swine manure can alter microbial community assembly and suppress emissions (Frazier et al., 2025). These biological approaches align with global priorities in sustainable agriculture by offering the potential to reduce environmental footprints, support animal health, and decrease reliance on chemical inputs (Jin Song et al., 2019).

Important benefits of microbiome-based odor control extend beyond the immediate goal of emission reduction. VOCs such as NH_3_ and hydrogen sulfide are respiratory irritants and are linked to stress responses and increased disease susceptibility in livestock (Cao et al., 2023). Furthermore, given the reported associations between malodorous waste environments and elevated pathogen loads, microbiome-driven odor mitigation strategies may also enhance on-farm biosecurity and reduce zoonotic disease risks (Abdugheni et al., 2023).

Beyond their environmental impact, odorous VOCs emitted from animal waste can also affect animal health, worker safety, and farm biosecurity (Lee et al., 2018; Wang et al., 2018). Among these compounds, NH_3_ and H_2_S are of particular concern in intensive livestock systems, where inadequate ventilation and manure accumulation can lead to elevated concentrations (Wang et al., 2018; Wu et al., 2021; Wu et al., 2020). Chronic exposure to NH3 is associated with respiratory epithelial damage, impaired mucociliary clearance, and increased susceptibility to respiratory infections in livestock (Mohammad Al-Kerwi et al., 2022; Barbosa et al., 2024; Faizah et al., 2024; Ameen et al., 2025). Hydrogen sulfide, although typically present at low concentrations, can reach hazardous levels during manure agitation and poses acute toxicological risks (Dai et al., 2015; Chou et al., 2016).

A deeper mechanistic understanding of microbiome modulation is essential not only for VOC mitigation but also for realizing secondary benefits such as improved herd health, immune function, and productivity. This review synthesizes current advances in microbiome engineering strategies targeting VOC emissions in animal waste systems. It focuses on the microbial mechanisms underlying odor formation and mitigation, evaluates evidence for practical on-farm application, and discusses the biological, technological, and economic challenges that constrain broader adoption. By integrating insights from gut microbiology, waste ecology, and applied microbial engineering, this review aims to clarify the realistic potential of microbiome-based approaches as components of comprehensive, sustainable waste management frameworks in animal production systems.

Literature for this narrative review was identified through searches of PubMed, Scopus, and SpringerLink using combinations of the following keywords: ‘odor mitigation’, ‘odor management’, ‘animal odor’, ‘volatile organic compounds’, ‘livestock waste’, ‘manure microbiome’, ‘probiotics’, ‘microbiome engineering’, ‘bioaugmentation’, ‘dietary crude protein’, ‘ammonia emissions’, ‘hydrogen sulfide’, ‘skatole’, ‘ruminant odor’, ‘swine manure’, and ‘feed additives’. The search focused on peer-reviewed articles published between 2015 and 2025, with priority given to recent original research and comprehensive reviews. Older foundational studies were included where they provided essential mechanistic background. Articles were selected based on their direct relevance to microbiome-driven VOC mitigation in livestock waste systems.

## 2 Origin of odorous volatile organic compounds in animal waste

VOCs emitted from animal waste are direct metabolic outputs of complex and dynamic microbial ecosystems (Joguet et al., 2023). Their production occurs along a biological continuum involving two interconnected stages: the gastrointestinal tract (GIT) of the host animal and the post-excretion environment of manure storage. The intensity and chemical profile of VOC emissions are ultimately determined by a four-way interaction between host physiology, diet composition, gut microbiota structure, and environmental conditions influencing microbial activity (Zhang et al., 2023).

### 2.1 The gut as a primary bioreactor

The anaerobic environment of the lower GIT serves as the primary biological site for generating odorous VOCs (Smith and Macfarlane, 1998). Here, the gut microbiota metabolizes dietary substrates into a diverse array of malodorous compounds, including NH_3_, phenols, indoles, sulfur-containing compounds, and VFAs (Diether and Willing, 2019). These metabolites originate from tightly interconnected microbial catabolic pathways that reflect substrate availability and microbial community composition.

Within the GIT system, a major source of malodor is the microbial fermentation of undigested dietary protein and amino acids in the hindgut. Proteins that escape host digestion in the small intestine are then hydrolyzed by extracellular proteases and peptidases secreted by proteolytic bacteria, yielding peptides and free amino acids (Diether and Willing, 2019; Wu et al., 2021). Prominent proteolytic taxa involved in this process in livestock include members of the genera *Bacteroides*, *Clostridium*, and *Streptococcus* (Pieper et al., 2016; Diether and Willing, 2019). Once internalized, amino acids undergo deamination, generating NH_3_ and carbon skeletons; additional NH_3_ is produced via urease−mediated hydrolysis of urea (Doelle, 2014; Diether and Willing, 2019) (Omer et al., 2025). While NH_3_ can be assimilated for microbial biosynthesis, excess production leads to its release into waste, contributing to pungent odors and potential toxicity (Diether and Willing, 2019).

It should be noted that the relative importance of these processes differs between monogastric and ruminant livestock (Tardiolo et al., 2025). In monogastric animals such as horses and poultry, substantial protein fermentation occurs in the hindgut when dietary proteins escape digestion in the small intestine, making this region a key source of odor-related metabolites. In contrast, ruminants possess a highly active foregut fermentation chamber (the rumen), where microbial degradation of dietary protein occurs prior to passage through the small intestine. NH_3_ produced in the rumen is often reutilized by rumen microbes for microbial protein synthesis or recycled via host urea transport mechanisms, which can alter the balance between nitrogen utilization and nitrogen excretion (Foggi et al., 2024). Consequently, the microbial pathways leading to NH_3_ formation and subsequent odor emissions may differ in magnitude and location across livestock species.

The carbon skeletons derived from amino acid catabolism are further metabolized by intestinal microbes into a variety of volatile compounds with strong odor activity (Diether and Willing, 2019). Aromatic amino acids are particularly important precursors. Microbial degradation of tryptophan produces indole and skatole, while tyrosine fermentation generates phenol and *p*-cresol (Sridharan et al., 2014; Diether and Willing, 2019). These compounds possess extremely low odor detection thresholds and are consistently identified as dominant contributors to livestock manure odor. In addition, fermentation of branched-chain amino acids such as leucine, isoleucine, and valine generates branched-chain fatty acids, including isovalerate, isobutyrate, and 2-methylbutyrate (Blachier et al., 2006; Omer et al., 2025). These metabolites serve as indicators of proteolytic fermentation and are associated with rancid or sweaty odor characteristics frequently reported in animal waste systems (Pellissery et al., 2020).

Sulfur-containing VOCs represent another critical class of malodorous compounds generated by gut microbial metabolism. Among these, hydrogen sulfide (H_2_S) and methanethiol are frequently reported as dominant contributors to livestock odor emissions (Diether and Willing, 2019). These compounds arise from microbial metabolism of both inorganic sulfur sources and sulfur-containing amino acids such as methionine and cysteine (Wu et al., 2021). Hydrogen sulfide is particularly problematic due to its extreme toxicity, corrosive properties, and characteristic rotten-egg odor that can be detected at very low concentrations (Arumugam et al., 2011).

In contrast to proteolytic fermentation, carbohydrate metabolism by saccharolytic bacteria primarily produces short-chain fatty acids, including acetate, propionate, and butyrate. These compounds are generally beneficial for gut health and host energy metabolism; however, their accumulation in manure and poorly aerated storage systems contributes to sour, pungent, and cheesy odors (Ganidi et al., 2009; Facchin et al., 2024). In the context of animal waste, an overabundance of fermentable substrate, combined with poor aeration in storage systems, can lead to an overproduction and accumulation of these VFAs. For instance, butyric acid is associated with rancid, cheesy, and vomit-like odors (Pellissery et al., 2020). The shift from a balanced gut or manure microbiome to one dominated by fermentative pathways, often due to dietary excess or environmental conditions, directly escalates the release of these acidic VOCs.

### 2.2 Host-microbiome-diet interactions and volatile organic compounds modulation

The specific VOC profile of animal waste is dynamic and depends on interactions between host dietary inputs and gut microbial metabolism (Omer et al., 2025). Excess dietary protein increases the flow of undigested nitrogenous substrates to the hindgut, favoring proteolytic fermentation. This metabolic shift results in elevated production of NH_3_, branched-chain fatty acids, phenols, and indoles (Omer et al., 2025). High-protein diets have also been shown to reduce overall microbial diversity and decrease beneficial short-chain fatty acids-producing bacteria, such as members of the *Roseburia* and *Eubacterium* groups, while increasing proteolytic and potentially pathogenic bacteria including *Escherichia coli* and *Clostridium* species (Wu et al., 2021; Omer et al., 2025). As discussed earlier, the location and magnitude of these processes differ between monogastric and ruminant livestock due to fundamental differences in gastrointestinal fermentation systems.

Conversely, adequate dietary fiber intake promotes saccharolytic fermentation and the production of short-chain fatty acids, which lower luminal pH and inhibit the activity of proteolytic and sulfate-reducing bacteria (Korpela, 2018). Through this mechanism, dietary fiber indirectly suppresses the formation of malodorous nitrogenous and sulfurous compounds (Korpela, 2018; Facchin et al., 2024). The underlying mechanism involves pH modulation. Saccharolytic fermentation of fiber produces short-chain fatty acids (acetate, propionate, butyrate), which lower luminal pH to approximately 5.5-6.5 (Xiang et al., 2019). This acidic environment directly inhibits the activity of proteolytic bacteria (e.g., Bacteroides, Clostridium) and sulfate−reducing bacteria (e.g., Desulfovibrio), which are pH−sensitive and thrive at neutral to slightly alkaline pH (Cho et al., 2015). Consequently, the production of NH_3_, H_2_S, and phenolic compounds is reduced, while beneficial short-chain fatty acids−producing taxa (e.g., Ruminococcaceae, Lactobacillus) are favored. Thus, the gut microbiome acts as a functional mediator that translates dietary composition into a characteristic fecal VOC signature, with balanced and diverse microbial communities favoring more complete and less odorous fermentation processes (Diether and Willing, 2019).

Host physiological status further modifies this interaction. Environmental stressors in livestock production systems, including elevated temperature and humidity, can disrupt gut microbial homeostasis and modify waste characteristics in swine and poultry (Guo et al., 2025). Although many microbial pathways responsible for VOC formation are conserved across mammals, the specific VOC profile, dominant microbial taxa, and emission intensity can vary substantially among livestock species due to differences in gastrointestinal physiology, diet, and manure management practices.

### 2.3 Distinct pathways in ruminants

Ruminants have unique VOC generation pathways in the rumen where microbial fermentation occurs prior to enzymatic digestion. Extensive microbial breakdown of fibrous plant material produces large quantities of VFAs as energy substrates for the host, along with methane (CH_4_) generated through archaeal methanogenesis. The major differences in microbial fermentation sites, dominant metabolic pathways, and resulting odor profiles between monogastric and ruminant livestock systems are summarized in **Table 1**.

**Table 1:** Comparative analysis of key volatile organic compound (VOC) generation pathways in monogastric and ruminant animals.

Feature	Monogastric (pigs, poultry)	Ruminant (cattle, sheep, goats)	Implication for mitigation
Primary VOC generation site	Undigested substrates undergo fermentation in the hindgut (Hoogeveen et al., 2020; Tardiolo et al., 2025).	Multi-chambered forestomach (rumen). Most fermentation occurs in the rumen (Li et al., 2022; Arya et al., 2024; Tardiolo et al., 2025).	Monogastrics: target hindgut via feed additives; Ruminants: target rumen via dietary manipulation
Key microbial process	Undigested protein fermentation → NH_3_, indoles, phenols (Pieper et al., 2016; Joguet et al., 2023; Noorman et al., 2025; Tardiolo et al., 2025).	Fiber fermentation → VFAs + methane; some protein fermentation (Belanche et al., 2023; Tardiolo et al., 2025).	Mitigation must address different primary substrates
Dietary influence on VOC precursors	High protein → increased N−excretion and odor (Jha et al., 2019).	High fiber → increased VFAs; protein source affects NH_3_ (Park et al., 2024a; Zhang et al., 2025).	Low crude protein diets effective in both; fiber type matters more in ruminants
Dominant VOC pathways & key compounds	NH_3_, H_2_S, skatole, p−cresol (Joguet et al., 2023)	VFAs (acetate, propionate, butyrate), methane, NH_3_ (in stored manure) (Arya et al., 2024; Tardiolo et al., 2025).	Odor control for ruminants must focus on manure storage as much as on animal
Mitigation strategy focus	Improve protein digestibility; probiotic consortia (Joguet et al., 2023).	Rumen modulation (tannins, essential oils); manure management (Park et al., 2024b).	Different product development pathways needed

Dietary protein source exerts a significant influence on rumen microbial composition and metabolic outputs. For example, in sheep, rapeseed meal supplementation has been associated with increased methane emissions and enrichment of methanogenic archaea such as *Methanobrevibacter*, whereas cottonseed meal supports higher abundances of *Lactobacillaceae* and results in reduced methane production (Wang et al., 2025). Despite extensive foregut fermentation, undigested substrates still reach the hindgut, where secondary fermentation processes contribute to the production of NH_3_, VFAs, and sulfur compounds that ultimately influence manure odor.

### 2.4 Post-excretion processes in manure storage

Following excretion, microbial activity continues within manure storage systems, constituting a second and distinct phase of VOC generation. The manure microbiome represents a dynamic ecosystem shaped by environmental parameters such as temperature, moisture, oxygen availability, and pH, and undergoes microbial succession over time (Sommer and Hutchings, 2001). Storage conditions often favor strict anaerobes, leading to sustained or increased emissions of hydrogen sulfide, NH_3_, and other reduced compounds (Fangueiro et al., 2015).

Microbial community composition and VOC production continue to evolve during storage, with evidence that diet-induced differences in the gut microbiome can persist post-excretion. For instance, the abundance of odor-associated genera such as *Weissella* and *Lactobacillus* in stored pig manure has been shown to reflect the dietary background of the host animal (Zhang et al., 2023).

### 2.5 Implications for microbiome-based mitigation

Understanding this two-stage biological origin of odor formation underscores the rationale for microbiome engineering. Effective interventions can target multiple points along the odor-generation continuum, including modulation of the gut microbiome through dietary optimization, probiotics, or feed additives to reduce precursor formation, as well as management of the manure microbiome via aeration, composting, or targeted bioaugmentation to redirect post-excretion metabolic pathways (Zhao and Kim, 2015; Zhang et al., 2023; Wang et al., 2025).

## 3 Microbiome engineering approaches for targeting VOCs in animal waste

Microbiome engineering for odor mitigation aims to reduce odorous VOCs by deliberately altering the composition and function of microbial communities at two critical points: the gastrointestinal tract of the host animal and the post-excretion manure environment (Park et al., 2024b; Chen et al., 2025). These biologically targeted strategies offer a sustainable alternative to conventional physical or chemical treatments by intervening directly in the core microbial pathways responsible for odor generation.

### 3.1 Probiotics and functional microbial consortia

Probiotic supplementation represents one of the most widely explored microbiome engineering strategies for odor mitigation. By introducing beneficial microorganisms, probiotics can competitively exclude odor-producing taxa, enhance saccharolytic fermentation, stabilize gut pH, and reinforce ecosystem resilience (Chen et al., 2025). However, single-strain interventions often yield inconsistent outcomes, prompting a shift towards multi-strain, functional microbial consortia that mimic natural ecosystems with complementary metabolic functions and greater functional redundancy (Park et al., 2024b).

Recent *in vivo* studies demonstrate significant, measurable odor reductions using designed consortia (Park et al., 2024b; Chen et al., 2025). For instance, a swine trial supplementing a consortium of *Bacillus subtilis* S14 and *B. amyloliquefaciens* S20 significantly reduced fecal skatole concentration, a potent indolic odorant with a low odor detection threshold (Chen et al., 2025). Similarly, a cattle study using a four-strain consortium (*B. subtilis, Lactobacillus acidophilus, L. casei, Saccharomyces cerevisiae*) in drinking water reduced manure emissions of NH_3_ (-64.1%) and dimethyl sulfide (-81.3%) over three months period (Park et al., 2024b).These reductions were associated with a microbiome shift favoring fiber-degrading taxa such as *Ruminococcaceae* and a concurrent decrease in odor-associated *Bacteroidota* (Park et al., 2024b).

The superior performance of microbial consortia is consistent with principles established in biodegradation research. Specifically, consortia achieve synergistic effects through three complementary mechanisms. First, cross−feeding: one strain degrades a complex substrate into intermediates that another strain further metabolizes, preventing accumulation of odor precursors (Smith et al., 2019). For example, Bacillus species producing proteases release amino acids that Lactobacillus species then ferment to lactate rather than to NH_3_ or skatole (Park et al., 2024b). Second, niche partitioning: different strains occupy distinct microhabitats (e.g., mucus layer vs. lumen), collectively suppressing odor−producing taxa more effectively than any single strain (Conti et al., 2020; Park et al., 2024b). Third, resilience to perturbations: functional redundancy within a consortium ensures that if one strain fails under stress (e.g., pH drop), others compensate, maintaining odor−suppressive activity. These principles guide the rational design of consortia for specific livestock systems (Liang et al., 2022). Additionally, biochemical cooperation improves overall metabolic efficiency and ecological stability, resulting in more predictable and durable outcomes (Zhang and Zhang, 2022). Key recent *in vivo* evidence supporting probiotic and consortium-based odor mitigation across livestock systems is summarized in **Table 2**.

**Table 2:** Recent *in vivo* evidence for probiotic and consortium efficacy.

Livestock	Probiotic/consortium strains	Key outcome (VOC reduction)	Reference
Swine	*Bacillus subtilis* S14 + *B. amyloliquefaciens* S20	Significant reduction in fecal skatole concentration.	(Chen et al., 2025)
Cattle	*B. subtilis* KNU-11, *Lactobacillus acidophilus* KNU-02, *L. casei* KNU-12, *Saccharomyces cerevisiae* KNU-06	NH_3_ (-64.1%), dimethyl sulfide (-81.3%), butyric acid (-84.6%).	(Park et al., 2024b)
Swine (litter)	Indigenous Microorganisms (IMO) consortium	NH_3_ emissions reduced by 29% and lowest odor intensity in sensory tests.	(Gobeil et al., 2025)

### 3.2 Dietary modulation and prebiotic strategies

Dietary intervention is a powerful indirect microbiome engineering tool, as diet dictates the substrates available for gut microbial metabolism (Burgos et al., 2010; Edouard et al., 2019; Katongole and Yan, 2022). Among dietary strategies, reducing excess crude protein while maintaining amino acid balance is one of the most robustly supported interventions for odor mitigation. This approach minimizes nitrogen excretion, thereby limiting the substrate pool available for microbial NH_3_ production. A controlled study in dairy cows demonstrated that average daily NH_3_ emissions from slurry increased linearly by 5.4 g for each 10 g/kg increase in dietary crude protein (from 141 to 201 g crude protein/kg DM) (Katongole and Yan, 2022). Similarly, in swine, reducing dietary crude protein by 3 percent units with appropriate amino acid supplementation decreased total nitrogen excretion by approximately 30% and ammonium nitrogen in slurry by 37%, leading to reductions in NH_3_ emissions of up to 50%. Complementary increases in fermentable dietary fiber further shift microbial metabolism toward saccharolytic pathways, enhancing short-chain fatty acid production and lowering intestinal pH. This acidic environment suppresses proteolytic and sulfate-reducing bacteria, thereby reducing the formation of nitrogenous and sulfur-containing VOCs (Chen et al., 2025). However, excessive crude protein restriction (below 12-13% for swine or 14-15% for dairy cattle) can limit essential amino acid supply, reducing microbial protein synthesis in ruminants or compromising growth and milk production in monogastrics (Esteves et al., 2021). Supplementation with crystalline amino acids (lysine, methionine, threonine) allows crude protein reduction without performance loss, as demonstrated in swine where 3 percentage point crude protein reduction with amino acid supplementation maintained growth while reducing NH_3_ emissions by 50% (Esteves et al., 2021). Three recent studies strongly support dietary approaches. Zeleke et al. (2025) demonstrated that reducing crude protein in dairy cow diets improved nitrogen-use-efficiency and reduced urinary nitrogen excretion without affecting rumen microbiome or cow performance. Another study showed that feeding laying hens low-protein diets supplemented with essential amino acids reduced nitrogen excretion by 19.7%, greenhouse gas emissions by 23.8%, and ammonia from manure composting by 29.2%, with no negative effects on egg production (Iio et al., 2025). This amino acid-balanced low- crude protein approach represents the current standard for diet-based odor mitigation (Cappelaere et al., 2021a). While dietary strategies are effective, they must be carefully optimized to avoid negative impacts on animal performance, health, and production efficiency. Successful implementation therefore requires a balance between nutritional adequacy and microbiome-driven environmental outcomes.

### 3.3 Bioaugmentation and post excretion microbiome manipulation

Engineering the microbiome of stored manure is crucial, as the gut and manure microbiome represents distinct ecosystems that continues to generate VOCs independently of the host. Bioaugmentation strategies involves adding microbial inoculants to manure to accelerate the degradation of odorous compounds. Among these approaches, micro-aeration has emerged as a particularly promising *in situ* intervention (Vu et al., 2022; Mutegoa and Sahini, 2023).

Micro-aeration involves the controlled introduction of small quantities of oxygen into otherwise anaerobic systems, such as manure storage tanks or anaerobic digesters, to regulate redox potential. This selectively inhibits sulfate-reducing bacteria responsible for hydrogen sulfide production while promoting sulfur-oxidizing bacteria that convert existing H_2_S into elemental sulfur (Vu et al., 2022). Micro-aeration has demonstrated H_2_S reduction efficiencies of 95-99% in laboratory and pilot−scale anaerobic digesters treating animal manure (Díaz et al., 2015; Fu et al., 2023; Froelich and Akdeniz, 2025) It is cost-effective and can be retrofitted into existing infrastructure (Vu et al., 2022; Mutegoa and Sahini, 2023). Likewise, 80-92% H_2_S removal during high-solids anaerobic digestion of poultry litter was reported when using sulfur-oxidizing bacteria with micro-aeration (Karumanchi et al., 2026). The methane yield improvements of 5-20% and costs of $0.0015-0.0045 per m³ biogas (Froelich and Akdeniz, 2025). This strategy exemplifies the convergence of microbiome engineering and waste management engineering.

### 3.4 Emerging precision microbiome approaches

Recent advances in multi-omics, systems biology, and computational modeling have enabled the emergence of precision microbiome engineering approaches that target specific metabolic functions rather than broad community shifts (Cummings et al., 2025). One such approach involves the rational design of synthetic microbial consortia assembled from first principles to achieve complementary and stable functions, such as complete nitrogen assimilation or targeted degradation of odor precursors. Another emerging strategy focuses on metabolic pathway inhibition, whereby specific enzymes involved in VOC production are selectively suppressed without disrupting beneficial microbial processes. In parallel, omics-guided diagnostics using metagenomic and metabolomic profiling allow real-time monitoring of microbial community dynamics and intervention efficacy, facilitating adaptive management strategies.

Despite their promise, the translation of genetically engineered systems faces regulatory and societal barriers (Cummings et al., 2025). Near−term progress will therefore rely on refining established approaches such as probiotic consortia, dietary optimization, and manure microbiome management. A comparative overview of microbiome engineering strategies, their mechanisms of action, target VOCs, and documented applications across livestock systems is provided in **Table 3**.

**Table 3:** Comparative analysis of microbiome engineering strategies for volatile organic compound (VOC) mitigation in animal waste management systems.

Approach category	Specific strategy	Mechanism	Primary target VOCs	Key evidence (species)	References
In-feed strategies (targeting gut microbiome)	Probiotic Consortia	Competitive exclusion; pH stabilization; enhanced saccharolytic fermentation.	NH_3_, H_2_S, skatole, VFAs	Cattle, swine	(Jin Song et al., 2019; Park et al., 2024b; Chen et al., 2025)
	Low Crude Protein (+ amino acids)	Reduces N−excretion; limits substrate for NH_3_ production	NH_3_	Swine, dairy cattle	(Lynch et al., 2007; Katongole and Yan, 2022)
	Prebiotics/Fiber	Shifts metabolism to saccharolytic; lowers gut pH	NH_3_, phenols, indoles.	Swine	(Lynch et al., 2007) (Park et al., 2024b)
Post-excretion strategies (targeting manure microbiome)	Bioaugmentation (constructed consortia	Alleviates NH_3_ inhibition; enhances methanogenesis	NH_3_, overall odor	Swine, dairy manure	(Yang et al., 2022)
	Microaeration	Inhibits sulfate−reducing bacteria; oxidizes H_2_S to S^0^	H_2_S	Swine Manure	(Di et al., 2025)
	Manure storage covers (Biocovers)	Creates surface layer oxidize gases	NH_3_, H_2_S, general odor	Swine	(Clanton et al., 2001)
Integrated & novel biological strategies	Synthetic consortia	Rational design with division of labor	Customizable	Concept)	Liang et al., 2022; Zhou and Zeeshan Ul Haq, 2025)

## 4 Health and biosecurity co-benefits of microbiome-based VOC mitigation

Microbiome-based strategies designed to reduce odorous VOC emissions may also provide important co-benefits for animal health and farm biosecurity (Lee et al., 2018; Wang et al., 2018). Important benefits of microbiome-based odor control extend beyond the immediate goal of emission reduction. VOCs such as NH_3_ and hydrogen sulfide are respiratory irritants and are linked to stress responses and increased disease susceptibility in livestock (Cao et al., 2023). Furthermore, given the reported associations between malodorous waste environments and elevated pathogen loads, microbiome-driven odor mitigation strategies may also enhance on-farm biosecurity and reduce zoonotic disease risks (Abdugheni et al., 2023).

Increasing evidence indicates that VOC production in livestock systems is closely linked to GIT microbial metabolism and the balance between proteolytic and saccharolytic fermentation pathways (Jin Song et al., 2019; Singh et al., 2022; Balasubramanian and Liu, 2024). Proteolytic bacteria such as *Clostridium* and *Escherichia*, not only generate odorous compounds but are also associated with intestinal inflammation and impaired gut barrier (Jin Song et al., 2019; Hitch et al., 2022; Singh et al., 2022). In addition to contributing to environmental odor emissions, these microbial metabolic pathways are frequently associated with intestinal inflammation and impaired gut barrier function. These microbial metabolic pathways are frequently associated with intestinal inflammation and impaired gut barrier function. In pigs, for example, high dietary protein intake increases colonic proteolytic bacteria (including E. coli and Clostridium perfringens) and is correlated with elevated luminal NH_3_, indoles, and phenols, as well as reduced colonic expression of tight junction proteins and increased inflammatory markers (Pieper et al., 2016; Wu et al., 2021).

Microbiome dysbiosis can increase intestinal permeability, allowing microbial products such as lipopolysaccharides (LPS) from Gram-negative bacteria to enter systemic circulation and trigger chronic low-grade inflammation (Hitch et al., 2022). This inflammatory state can negatively affect immune function and increase host susceptibility to pathogens (Hitch et al., 2022). Conversely, a balanced gut microbiome dominated by saccharolytic fermentation promotes the production of beneficial metabolites, particularly short-chain fatty acids such as butyrate (Hitch et al., 2022; Singh et al., 2022). These metabolites support intestinal epithelial integrity, regulate immune responses, and contribute to colonization resistance against pathogenic microbes (Laursen et al., 2021; Lopez-Tello et al., 2022).

**Figure 1:**
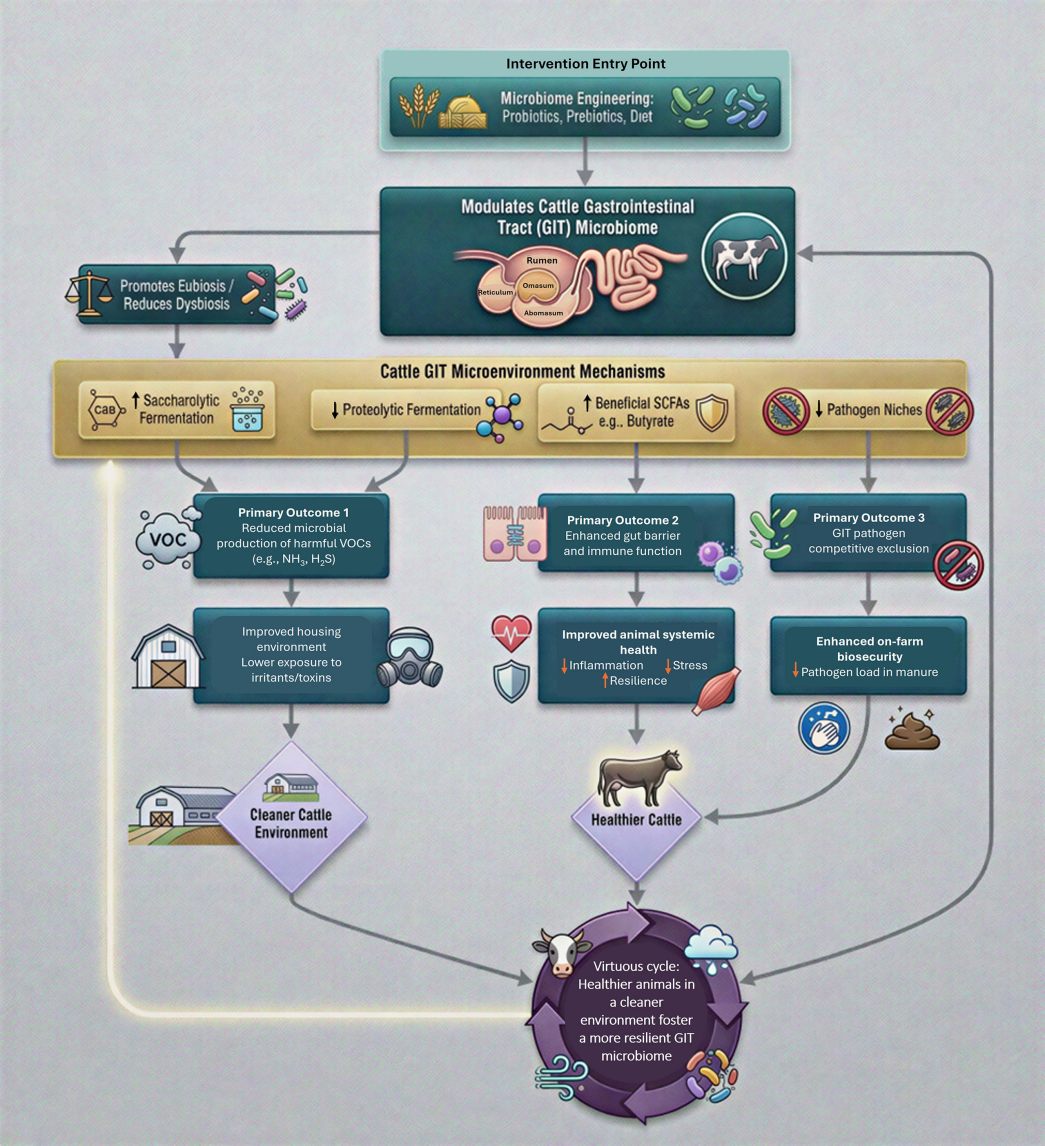
Conceptual framework illustrating how microbiome engineering for volatile organic compound (VOC) reduction can simultaneously improve animal health and farm biosecurity. The cycle begins with interventions (e.g., probiotics, dietary modulation) that promote eubiotic GIT state, characterized by a shift toward saccharolytic fermentation and increased production of beneficial metabolites like short-chain fatty acids (SCFAs). This shift yields three primary outcomes: (1) reduced microbial production of harmful VOCs (e.g., NH_3_, H_2_S); (2) enhanced gut barrier function and immune regulation; and (3) competitive exclusion of intestinal pathogens. Collectively, these lead to a cleaner housing environment and healthier, more resilient animals. Critically, these endpoints create a self-reinforcing virtuous cycle (denoted by the central arrow), where a lower environmental stress load and a more robust host physiology further stabilize the beneficial gut microbiome, ensuring the long-term sustainability of the intervention.

Microbiome engineering approaches, including dietary modulation, probiotics, and microbial consortia, can therefore deliver dual benefits by simultaneously reducing the microbial generation of odorous VOCs and promoting host health. As illustrated in **Figure 1**, these processes form a self-reinforcing “virtuous cycle,” in which improved GIT microbial balance reduces proteolytic fermentation and VOC production while strengthening gut barrier function and immune resilience (Jin Song et al., 2019). The resulting reduction in environmental VOC burden may further alleviate physiological stress in animals and improve housing conditions, contributing to more resilient and biosecure production systems (Hitch et al., 2022; Singh et al., 2022). The causal chain operates as follows: Lower airborne NH_3_ and H_2_S concentrations reduce oxidative stress and inflammation in the respiratory epithelium (Wang et al., 2019). This, in turn, decreases systemic inflammatory mediators (e.g., IL-6, TNF-α) that can compromise gut barrier integrity (Zhao and Kim, 2015). An intact gut barrier prevents translocation of luminal LPS and pathogens, thereby reducing immune activation and allowing the host to allocate more resources to growth and reproduction (Kebreab et al., 2016). Conversely, high NH_3_ exposure (≥10 ppm in poultry, ≥7 ppm in swine) has been shown to increase intestinal permeability and reduce expression of tight junction proteins (claudin-1, occludin) (Belloir et al., 2017; van Harn et al., 2019). Thus, microbiome interventions that lower VOCs at source indirectly protect gut health, creating a positive feedback loop.

### 4.1 Enhanced farm biosecurity through microbial niche management

Manure storage systems are not only sources of odor but also critical reservoirs for zoonotic pathogens (e.g., *Salmonella*, *E. coli*) and hotspots for antimicrobial resistance (Arjmand et al., 2025). The anaerobic, nutrient-rich conditions that favor VOC production also support the persistence and proliferation of these pathogens. Microbiome engineering strengthens biosecurity through the principle of competitive exclusion (Buffie and Pamer, 2013; Jin Song et al., 2019). A diverse, functional, and stable microbial community in the gut and manure occupies ecological niches, consumes available resources, and produces inhibitory compounds, making it more difficult for pathogens to establish (Jin Song et al., 2019). For instance, probiotic *Bacillus* strains have demonstrated direct antagonistic effects against *Salmonella* growth (Stern et al., 2001). while post-excretion bioaugmentation with tailored microbial consortia accelerate the safe decomposition of manure, shortening pathogen survival times (Liu et al., 2025; Priyadi et al., 2025).

Mathematical modeling underscores that no single biosecurity measure is sufficient to prevent outbreaks; integrated strategies are essential (Arjmand et al., 2025). Reducing the pathogen load at its source via gut microbiome modulation is a foundational, preventive layer of defense. When combined with manure management and strict operational hygiene, it forms a comprehensive One Health approach that mitigates the risk of zoonotic spillover to farmworkers and the community, while also reducing the need for therapeutic antibiotics and the associated antimicrobial resistance pressure (Arjmand et al., 2025).

### 4.2 Contributions to productivity and sustainable livestock systems

The cumulative benefits of microbiome-based VOC mitigation translate into tangible gains for sustainable production. Improved respiratory and systemic health directly enhances animal productivity, including better feed conversion ratios, higher growth rates, and improved reproductive performance (Morgado et al., 2023; Dock, 2024). Stress reduction leads to more stable and natural animal behavior, which is intrinsically linked to welfare and can improve product quality (Acharya et al., 2022; Kannan and Miranda-de la Lama, 2023).

From a sustainability perspective, source reduction of VOCs aligns with circular economy principles. It is more efficient and often more economical than treating emissions after they are produced. Strategies like dietary optimization and probiotic use integrate seamlessly into existing farm management, reducing reliance on costly end-of-pipe remediation technologies (Hitch et al., 2022; Dock, 2024). Furthermore, by lowering the environmental footprint of livestock operations, reducing atmospheric pollution, nutrient runoff, and odor complaints, these approaches improve social license to operate and help meet increasingly stringent environmental regulations (Wu et al., 2020).

Ultimately, viewing odor mitigation through the lens of microbiome engineering reframes it from a waste management problem to a core component of preventive veterinary medicine and sustainable farm system design. The goal shifts from masking odors to cultivating an internal and external microbial ecosystem that supports animal health, safeguards public health, and enhances environmental stewardship.

## 5 Practical challenges and scientific limitations in translating microbiome engineering to the farm

The promise of microbiome engineering for sustainable odor mitigation is anchored in robust biological principles. However, translating promising experimental findings into reliable, cost-effective, on-farm applications remains constrained by a convergence of scientific, technical, and practical barriers. These challenges stem from the inherent complexity of biological systems, significant variability in farm environments, and gaps in our ability to predict and control microbial community outcomes at scale (Yan et al., 2024). Addressing these limitations is critical for developing robust, next-generation odor mitigation and waste management strategies.

### 5.1 Technical and methodological hurdles: from measurement to prediction

A foundational challenge is accurately defining the problem itself. Odor is a sensory perception, not directly quantified by measuring single gases like NH_3_. While NH_3_ and H_2_S are often the most concentrated gases, their contribution to nuisance odor is dwarfed by compounds with much lower olfactory thresholds, such as trimethylamine, butyric acid, and various aldehydes (Ren et al., 2025). A meta-analysis revealed that despite high concentrations, NH_3_’s odor contribution can be minimal compared to sulfides or VFAs when assessed via the Odor Activity Value, which weights concentration by human perception threshold (Ren et al., 2025). Therefore, interventions that successfully lower NH_3_ may not meaningfully reduce perceived odor if other key odorants (e.g., volatile sulfur compounds) are unaffected, leading to a misalignment between engineering targets and practical outcomes.

Furthermore, the dynamic nature of waste systems makes efficacy measurement and prediction difficult. Manure VOC profiles are not static; they change dramatically with animal age, diet, health status, and management events (Haider et al., 2024). For instance, poultry manure VOC signatures shift significantly after vaccinations, a common husbandry stressor, demonstrating how host physiology directly and rapidly alters the metabolic output targeted for engineering (Haider et al., 2024). Traditional linear models struggle to account for these complex, non-linear interactions between diet, herd size, climate, and microbial activity (Ouatahar et al., 2024). Emerging artificial intelligence (AI) and neural network models show promise in modeling this complexity to predict waste generation and emissions, but they require extensive, high-quality farm-specific data for training and validation, which is often lacking (Tryhuba et al., 2025).

### 5.2 Biological complexity: microbial and contextual variability

The core assumption of microbiome engineering, that introduced microbes will predictably alter community function, collides with the resilience and variability of native ecosystems. Microbial communities in the gut and manure are shaped by intense competition and niche specialization. Introduced probiotic strains or bioaugmentation consortia often fail to establish or persist due to colonization resistance from the resident microbiome (Caballero-Flores et al., 2020; Chen et al., 2024). Colonization resistance arises because the resident microbiome occupies available metabolic niches, consumes limiting nutrients, and produces bacteriocins or other inhibitory compounds that exclude invaders (Liang et al., 2022; Park et al., 2024b). For a probiotic strain to establish, it must either (i) occupy a niche not already filled (e.g., a novel metabolic function), (ii) be administered at very high doses to temporarily overwhelm residents, or (iii) be co-administered with a prebiotic that selectively supports its growth (Cappelaere et al., 2021b; Liang et al., 2022). Even when transient establishment occurs, the strain’s metabolic activity (e.g., VOC degradation) may diminish after supplementation ceases. This explains why some probiotics show efficacy only during continuous administration, whereas prebiotic fibers that shift the native microbiome can produce more durable effects. Understanding these ecological principles is essential for designing interventions with lasting impact (Cappelaere et al., 2021b; Park et al., 2024a). The survival and function of these strains are further challenged by environmental stressors within barns and manure storage systems, such as pH shifts, temperature fluctuations, and the presence of antimicrobial agents (Wendel, 2021; Zielińska et al., 2025).

Effectiveness is highly dependent on species, production stage, and manure management system, with no universal solution (Ambrose et al., 2023; Yan et al., 2024) (**Table 4**). Regional differences in climate, feed, and genetics further hinder off−the−shelf product development (Tryhuba et al., 2025).

**Table 4:** Key sources of variability influencing the applicability, effectiveness, and challenges of microbiome engineering strategies for odor mitigation in livestock production systems.

Source of variability	Impact on microbiome engineering strategy	Example/consequence
Livestock species	Determines primary fermentation site (rumen vs. hindgut)	Ruminants: target rumen methanogens; Monogastrics: target colonic proteolytic bacteria
Manure management stage	Creates distinct physicochemical environments (aerobic/anaerobic, solid/liquid).	In−feed additives work across stages; bioaugmentation needs anaerobic conditions
Diet and feeding regimen	Supplies substrates for VOC production (protein, fiber, additives)	High-protein diets increase NH_3_ potential; may overwhelm probiotic effects.
Farm scale & infrastructure	Influences feasibility of uniform intervention application and manure handling.	Large-scale slurry: bioaugmentation possible; small solid systems: feed−based only
Climate and season	Alters microbial activity and emission rates	Higher temperatures increase VOC production; may require seasonal adjustment of interventions

### 5.3 Risk of unintended consequences and trade-offs

Altering complex microbial ecosystems carries inherent risks of unintended outcomes, which must be proactively assessed.

#### 5.3.1 Pollution swapping and metabolic trade-offs

Suppressing one microbial pathway can inadvertently enhance another. A prime example is the trade-off between NH_3_ and nitrous oxide (N_2_O), a potent greenhouse gas (Sokolov et al., 2020). Techniques like manure acidification or shallow injection can dramatically reduce NH_3_ volatilization but may increase N_2_O emissions under certain conditions (Sokolov et al., 2020; Sigdel et al., 2024). Similarly, strategies that inhibit sulfate-reducing bacteria to control H_2_S could redirect sulfur or carbon flow to other odorous or problematic metabolites (Karnachuk et al., 2021; Qi et al., 2025).

#### 5.3.2 Antimicrobial resistance and pathogen dynamics

Livestock manure is a recognized reservoir of antimicrobial resistance genes and zoonotic pathogens (Alem et al., 2025; Trinchera et al., 2025). The selective pressure exerted by some feed additives or the horizontal gene transfer within engineered microbial communities could potentially exacerbate the spread of antimicrobial resistance genes. For instance, probiotic Bacillus strains used in livestock have been shown to carry and potentially transfer tetracycline resistance genes under co−selective pressure (Guan et al., 2025). Furthermore, manure from animals fed certain antimicrobial growth promoters has been associated with elevated antimicrobial resistance genes abundances in soil and water (He et al., 2020; Napit et al., 2025). While some probiotics aim to exclude pathogens, a poorly understood or unstable intervention might disrupt the ecological balance that naturally suppresses pathogens, creating new biosecurity risks (Black et al., 2021; Chen et al., 2024).

#### 5.3.3 Impact of co-occurring agrochemicals

Farm environments contain complex chemical mixtures, including pesticides, disinfectants, and veterinary antibiotics (Saeed et al., 2024; Kweon et al., 2025). Many of these “non-antimicrobial” chemicals have understudied antimicrobial properties that can damage host-associated microbiomes (Lagadinou et al., 2020). An engineered probiotic strain could be rendered ineffective or its introduction could have unpredictable consequences in the presence of these chemicals, adding a layer of uncertainty to real-world application (Upadhayay et al., 2023; Davran Bulut and Celebioglu, 2024).

### 5.4 Barriers to adoption: regulation, economics, and proof

Even a scientifically sound intervention faces steep barriers to commercial adoption. Fragmented regulatory frameworks (feed additive vs. biologic vs. environmental amendment) and divergent approval pathways across jurisdictions (EU vs. U.S.) increase development costs and discourage investment (Spacova et al., 2023; Mârza et al., 2025) For farmers, any new technology must demonstrate a clear return on investment. The economic benefits of odor reduction, such as improved community relations or reduced risk of regulatory action, are often intangible or long-term. Convincing evidence requires large-scale, longitudinal on-farm trials that control immense variability. Currently, many studies are short-term, small-scale, or conducted in controlled research settings, limiting confidence in their commercial practicality (Yan et al., 2024; Mârza et al., 2025). Furthermore, successful implementation requires seamless integration into daily farm operations without demanding major changes to housing, feeding, or manure handling infrastructure. Interventions that are labor-intensive, require specialized equipment, or interfere with other management priorities are unlikely to be adopted.

## 6 Future directions and research priorities for translation and impact

**Figure 2:**
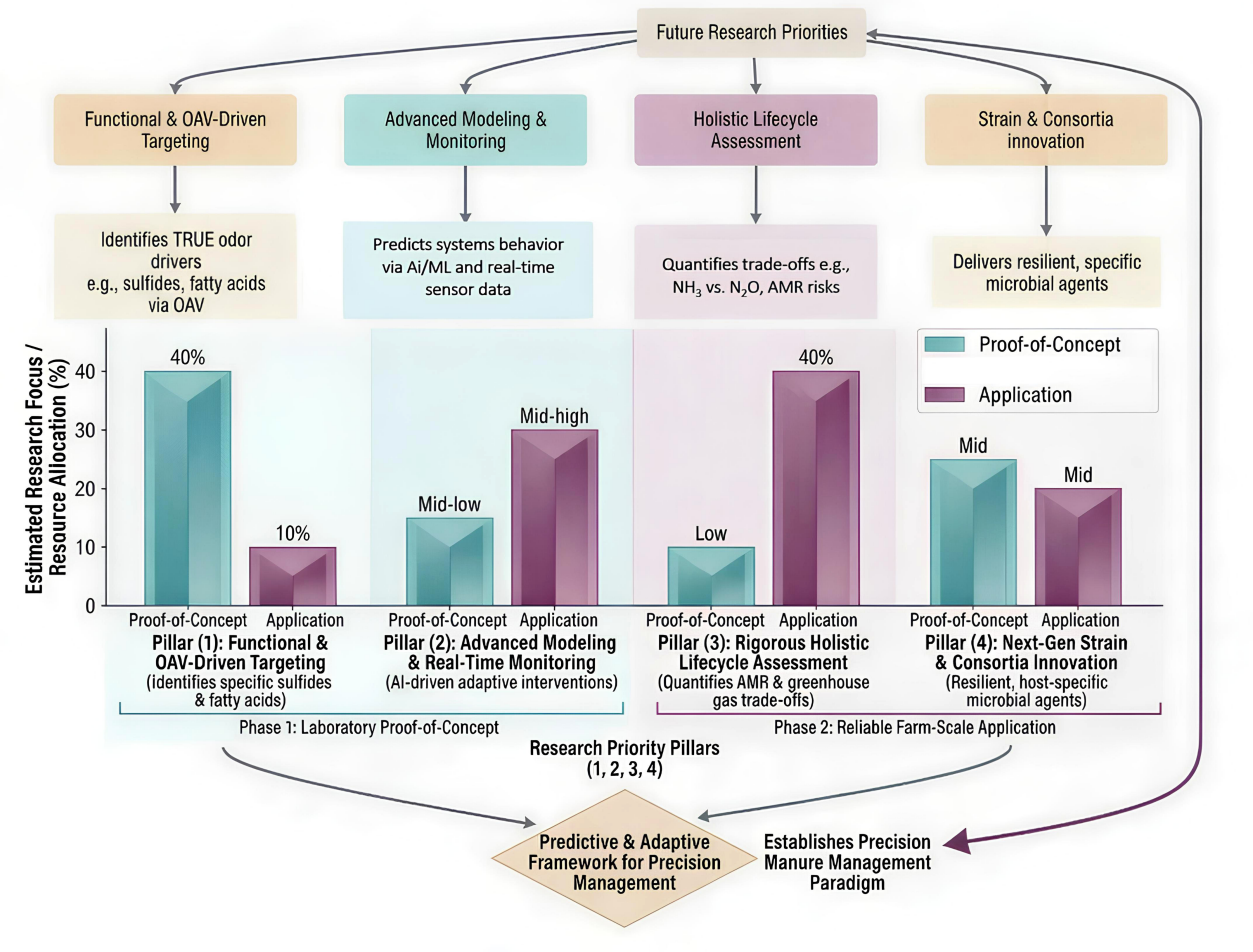
Integrated research priority framework for advancing microbiome-based VOC mitigation toward precision manure management. This conceptual framework defines four interconnected research pillars required to translate microbiome-driven volatile organic compound (VOC) mitigation from laboratory proof-of-concept to reliable farm-scale applications. Under the umbrella of future research priorities, the framework progresses across two implementation phases. *In Phase 1* (Laboratory proof-of-concept), emphasis is placed on (1) Functional and Odor Activity Value (OAV)-driven targeting, which identifies the true odor-driving compounds (e.g., sulfides and volatile fatty acids) beyond traditionally monitored gases, and (2) Advanced modeling and real-time monitoring, integrating artificial intelligence (AI), machine learning (ML), and sensor-based systems to predict microbial and emission dynamics and enable adaptive intervention strategies. *In Phase 2* (Reliable farm-scale application), priorities shift toward (3) Rigorous holistic lifecycle assessment, which quantifies environmental and biological trade-offs, including NH_3_ (NH_3_), nitrous oxide (N_2_O), and antimicrobial resistance (AMR) risks, and (4) Next-generation strain and consortia innovation, focused on developing resilient, host-specific microbial solutions with consistent performance under field conditions.

Translating microbiome engineering from a promising concept to a reliable, on-farm odor mitigation technology necessitates a coordinated research agenda. Based on the gaps identified in this review, we propose five priority areas for future research (summarized in **Table 5** and illustrated in **Figure 2**). Each priority is framed as a specific, actionable direction.

**Table 5:** Future research directions and translational priorities for microbiome-based odor mitigation in livestock and waste management systems.

Research direction	Core statement	Supporting evidence	Key references
1. Functional & mechanism-driven research	Future work must employ multi-omics to identify functional genes and metabolic networks, moving beyond taxonomy	• Metagenomic studies are already mapping microbial succession (*Lactobacillus* → methanogenic archaea) and key pathways like aromatic compound degradation in waste systems. This functional approach is critical for optimization. • Odor Activity Value identifies that major odorants are often trimethylamine and butyric acid, not just the most concentrated gases like ammonia.	(Akinsola et al., 2025; Ren et al., 2025)
2. Longitudinal & farm-scale validation	Strategies require validation under real farm conditions across seasons and production cycles to prove durability and economic viability	• Probiotic efficacy is highly context-dependent (diet, age, genetics, management practices. • Reviews call for more field-based validation and long-term assessments of productivity and environmental impact to translate research into practice.	(Idowu et al., 2025).
3. Integration with precision livestock farming (	Integrating with sensors, real-time monitoring, and AI enables dynamic, data-driven management of microbiome interventions	• Precision livestock farming uses information technology for real-time monitoring to improve health, welfare, and environmental impact • Deep learning and machine learning represent the forefront of precision livestock farming research, ideal for analyzing complex microbiome and sensor data. • Electronic noses and other sensors are established tools for odor measurement and can be integrated into such systems.	(Jiang et al., 2023; Ren et al., 2025).
4. Regulatory harmonization and responsible innovation	Clear, harmonized regulatory frameworks and risk assessments are needed to commercialize microbiome products responsibly	• Regulatory frameworks for probiotics in animal feed vary widely (EU, US, Asia), complicating global commercialization. • Risk assessments must consider that animal microbiomes are reservoirs for Antimicrobial Resistance Genes; interventions should avoid promoting their spread. • A “One Health” approach (human, animal, environment) is essential for responsible innovation.	(Leistikow et al., 2022; Mârza et al., 2025; Napit et al., 2025)
5. Integrated Circular Bioeconomy Systems	Microbiome engineering should be a component of circular systems that convert waste into resources like biogas and recovered nutrients.	• Anaerobic digestion for biogas production is a commercially established example of using complex microbial communities to valorize waste. • The circular bioeconomy framework transitions from linear waste models to sustainable, biobased cycles, with microbes playing a central role. • Optimizing these microbial processes through engineering directly supports this sustainable transition.	(Antranikian and Streit, 2022; Akinsola et al., 2025; Troiano and Studer, 2025)

### 6.1 Functional and mechanism−driven targeting of odor activity value

Future research must move beyond measuring bulk gases (e.g., NH_3_) and instead identify the specific VOCs that drive sensory nuisance using odor activity value analysis. Multi−omics approaches (metagenomics, metatranscriptomics, metabolomics) should be employed to link microbial functional genes and metabolic pathways to the production of key odorants such as sulfides, volatile fatty acids, and indoles. This mechanistic understanding will enable rational design of targeted interventions rather than trial−and−error approaches.

### 6.2 Longitudinal, farm−scale validation under real-world conditions

Most current evidence derives from short−term, small−scale, or controlled environment studies. Future research must prioritize long−term (≥1 year), farm−scale trials that account for seasonal variation, dietary changes, animal health fluctuations, and manure management practices. These studies should measure not only VOC reduction but also animal performance, economic return on investment, and farmer acceptability to bridge the gap between proof of concept and commercial adoption.

### 6.3 Integration with precision livestock farming and real−time monitoring

Advances in sensor technology (electronic noses, gas detectors) and artificial intelligence/machine learning offer the potential for dynamic, data−driven management of microbiome interventions. Future work should develop integrated precision livestock farming systems that continuously monitor VOC emissions, predict odor episodes, and trigger targeted interventions (e.g., automated probiotic dosing or micro−aeration). This requires collaboration between microbiologists, engineers, and data scientists.

### 6.4 Rigorous lifecycle assessment to avoid unintended trade−offs

Before scaling any microbiome engineering strategy, holistic lifecycle assessments must be conducted to identify potential pollution swapping or unintended consequences. Specific priorities include quantifying the effects of NH_3_ reduction strategies on N_2_O emissions (greenhouse gas), assessing the impact of interventions on antimicrobial resistance gene dissemination, and evaluating the fate of engineered microbes in the environment. These assessments should follow standardized protocols to enable cross−study comparisons.

## 7 Conclusion

Translating microbiome engineering into practical on-farm odor mitigation requires a shift toward precision manure management. This means moving beyond measuring dominant gases like NH_3_ and instead using tools such as odor activity value to identify the volatile compounds that drive sensory nuisance. Robust management will depend on advanced modeling and real-time monitoring; emerging AI and neural network models, fed by electronic nose data, can predict emissions and guide interventions. Before scaling any strategy, rigorous lifecycle assessments are essential to avoid unintended trade-offs, such as reducing NH_3_ volatilization only to increase nitrous oxide emissions. Finally, overcoming biological inconsistency demands next-generation microbial innovations, synthetic consortia and probiotic strains with engineered resilience, host-specificity, and well-defined mechanisms of action. Microbiome engineering offers a biologically grounded approach to targeting VOCs from animal waste, addressing odor at its microbial source rather than through symptomatic control. Current evidence supports dietary modulation, probiotic and consortium-based interventions, and post-excretion microbiome manipulation, though outcomes remain highly context-dependent. Significant challenges persist, including microbial stability, system-specific variability, and a lack of long-term, farm-scale validation. Based on the evidence synthesized in this review, the following recommendations are offered. First, researchers and practitioners should adopt odor activity value targeting to move beyond measuring ammonia alone and instead identify the low−threshold volatile organic compounds (sulfides, volatile fatty acids, skatole) that drive actual sensory nuisance. Second, long−term, farm−scale validation trials (≥1 year) are urgently needed to assess efficacy across seasons, dietary changes, and manure management systems, while also documenting economic return on investment. Third, integration with precision livestock farming tools, including electronic noses and artificial intelligence−based models, would enable real−time monitoring and adaptive intervention. Fourth, before scaling any strategy, holistic lifecycle assessments must quantify potential pollution swapping (e.g., reduction of NH_3_ increasing N_2_O emissions) and evaluate risks related to antimicrobial resistance gene dissemination. Fifth, next−generation microbial innovations should focus on rationally designed synthetic consortia with defined metabolic functions and strains engineered for resilience to on−farm stressors such as pH shifts, temperature fluctuations, and antimicrobial residues. When applied judiciously and supported by robust evidence, microbiome engineering can meaningfully improve air quality, animal welfare, and the sustainability of livestock production.
